# Effects of short‐term cognitive‐coping therapy on resting‐state brain function in obsessive‐compulsive disorder

**DOI:** 10.1002/brb3.2059

**Published:** 2021-02-09

**Authors:** Jian‐Dong Ma, Chang‐Hong Wang, Ping Huang, Xunan Wang, Li‐Jing Shi, Heng‐Fen Li, De‐En Sang, Shao‐Jie Kou, Zhi‐Rong Li, Hong‐Zeng Zhao, Hong‐Kai Lian, Xian‐Zhang Hu

**Affiliations:** ^1^ Xinxiang Medical University Affiliated Second Hospital Xinxiang Henan P. R. China; ^2^ The Fifth People's Hospital of Kaifeng Kaifeng Henan P. R. China; ^3^ Zhengzhou University First Affiliated Hospital Zhengzhou Henan P. R. China; ^4^ Workstation of Henan Province for Psychiatry experts Kaifeng Henan P. R. China; ^5^ Zhengzhou University Affiliated Zhengzhou Central Hospital Zhengzhou P. R. China

**Keywords:** cognitive‐coping therapy, obsessive‐compulsive disorder, OCD, psychotherapy, rs‐fMRI, treatment

## Abstract

**Background:**

Obsessive‐compulsive disorder (OCD) tends to be treatment refractory. Recently, cognitive‐coping therapy (CCT) for OCD is reported to be an efficacious psychotherapy. However, the underlying neurophysiological mechanism remains unknown. Here, the effects of CCT on OCD and the resting‐state brain function were investigated.

**Methods:**

Fifty‐nine OCD patients underwent CCT, pharmacotherapy plus CCT (pCCT), or pharmacotherapy. Before and after a 4‐week treatment, Yale‐Brown obsessive‐compulsive scale (Y‐BOCS) was evaluated and resting‐state functional magnetic resonance imaging (rs‐fMRI) was scanned.

**Results:**

Compared with the baseline, significant reduction of Y‐BOCS scores was found after four‐week treatment (*p* < .001) in groups of CCT and pCCT, not in pharmacotherapy. Post‐treatment Y‐BOCS scores of CCT group and pCCT group were not different, but significantly lower than that of pharmacotherapy group (*p* < .001). Compared with pretreatment, two clusters of brain regions with significant change in amplitude of low‐frequency fluctuation (ALFF) were obtained in those who treated with CCT and pCCT, but not in those who received pharmacotherapy. The ALFF in cluster 1 (insula, putamen, and postcentral gyrus in left cerebrum) was decreased, while the ALFF in cluster 2 (occipital medial gyrus, occipital inferior gyrus, and lingual gyrus in right hemisphere) was increased after treatment (corrected *p* < .05). The changes of ALFF were correlated with the reduction of Y‐BOCS score and were greater in remission than in nonremission. The reduction of the fear of negative events was correlated to the changes of ALFF of clusters and the reduction of Y‐BOCS score.

**Conclusions:**

The effectiveness of CCT for OCD was related to the alteration of resting‐state brain function—the brain plasticity.

**Trial Registration:**

ChiCTR‐IPC‐15005969.

## Graphical abstract

This study investigated the effects of CCT on OCD and the resting‐state brain function were investigated in 59 OCD patients underwent CCT, pharmacotherapy plus CCT (pCCT), or pharmacotherapy. The effectiveness of CCT for OCD was related to the alteration of resting‐state brain function ‐ the brain plasticity.

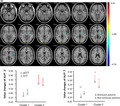

## 1 INTRODUCTION

Obsessive‐compulsive disorder (OCD), one of chronic and debilitating mental disorders, affects 2%‐3% of the United States and the Chinese populations (Holes et al., 2014; Huang et al., 2019) and tends to be treatment refractory. Current first‐line treatments for OCD, serotonin reuptake inhibitors (SRIs) and cognitive‐behavioral therapy (CBT), are partially effective (Hollander et al., 2002). Less than 25% of patients achieve minimal symptoms from SRIs (Bjorgvinsson et al., 2007), or 50% from CBT after 8 weeks of exposure and response prevention (ERP) and 6‐month maintenance treatment achieve minimal OCD symptom (Foa et al., 2015). Patients usually relapse after they stop taking the medication (Hollander et al., 2003; Koran et al., 2002). Despite that CBT benefits 60%–70% of treatment completers with OCD following a course of ERP, more than 70% of treatment completers are left with residual symptoms (Rufer et al., 2005). About 35% of the patients do not respond to the treatment recommended by current guidelines in terms of a reduction of symptom severity of at least 35% (Kulz et al., 2014). Relapse rates three months after discontinuation of intensive CBT are up to 50% (Simpson et al., 2005). Almost 60% of patients with OCD are considered to be refractory to pharmacotherapy, and up to 40% of patients with OCD are refractory to ERP (Bjorgvinsson et al., 2007; Foa, 2010). Furthermore, it usually takes more than 12 weeks to achieve significant clinical response when treating OCD patients with the pharmacotherapy or CBT (Foa, 2010; Math & Janardhan Reddy, 2007). The failure to respond to treatment may reflect the nature of the treatment received (Krebs et al., 2015) and/or the nature of OCD.

Recently, cognitive‐coping therapy (CCT) (Hu, 2010; Hu & Ma, 2011; Hu et al., 2012, 2015; Ma et al., 2013; Sang et al., 2020) for OCD has been developed. In CCT a fear of negative events (e.g., fear of contaminations, fear might harm oneself or others), obsessions are considered to be stressors, and compulsions to be responses to the stressors. The effects of these stressors on compulsions will be reduced when proper coping strategies are used (Hu et al., 2012). CCT has been reported to achieve higher response and remission rates, lower relapse rate and drop‐off rate in a 12‐month follow‐up, and higher level of social‐occupational function. CCT has the similar efficacy in drug‐resistant versus nondrug‐resistant OCD (Ma et al., 2013) and overt versus covert compulsions (Hu et al., 2015). However, neurophysiological mechanism related to the efficacy of CCT for OCD remains largely unknown.

Previously, the brain mediation of response to CBT in OCD has been investigated but the results are not consistent. Positron emission tomography (PET) scans on 10 OCD patients after four‐week intensive CBT demonstrates that significant bilateral decreases in normalized thalamic metabolism but a significant increase in right dorsal anterior cingulated cortex activity (Saxena et al., 2009). Another PET scans on 18 OCD patients before and after 8–12 weeks of CBT show that the patients who responded to CBT showed significant decreases in normalized right caudate glucose metabolism (Baxter et al., 1992). Since the therapeutic strategies are different between CCT and CBT, it is rational to assume that the brain mediation of response to CCT differs from that to CBT. Recently, resting‐state functional magnetic resonance imaging (rs‐fMRI) is increasingly used to understand the brain function at resting state in psychiatric disorders. To investigate potential neurophysiologic mechanism underlying the CCT for OCD, rs‐fMRI has the advantage of identifying neurophysiologic mechanisms that are not specific to the task employed, which may lead to controversial results. For example, while dysfunction in orbital, medial frontal, and striatal areas are reported to contribute to the pathogenesis of OCD, other evidence indicates there is broader cortical dysfunction in the disorder (Stern et al., 2012). The rs‐fMRI focuses on spontaneous low‐frequency fluctuations (<0.1 Hz) in the BOLD signal to detect the brain rest network (Lee et al., 2013). Also, amplitude of low‐frequency fluctuation (ALFF) is gradually accepted to investigate the mental disorders including OCD (Hou et al., 2012; Reggente et al., 2018; Zang et al., 2007) and is reported to have excellent discrimination between drug‐naïve OCD and healthy controls (Bu et al., 2019). ALFF is one of rs‐fMRI parameters and a representation of regional spontaneous neuronal activity. ALFF measures the deviation, rather than the mean of a period, of BOLD. Therefore, it is not a parameter of hyper‐ or hypo‐activation; it represents regional spontaneous neuronal activity.

This study sought to investigate the potential neurophysiologic mechanism underlying the effectiveness of short‐term CCT using the rs‐fMRI in a before and after study among OCD patients who received pharmacotherapy, pharmacotherapy plus CCT (pCCT), and CCT only. It was hypothesized that short‐term CCT would effectively reduce the severity of symptoms and that the effectiveness was related to the alteration of resting‐state brain activities involving in fear processing and/or cognitive‐emotional processing, such as insula.

## 2 METHODS

### 2.1 Participants

Participants were recruited from the Second Hospital of Xinxiang Medical University and referred by the medical centers located in Zhengzhou City and Kaifeng City, Henan Province from May 2013 to April 2015. All patients met DSM‐IV diagnostic criteria for OCD. All potential recruits undertook a semi‐structured clinical interview to screen for current axis‐I disorders. Individuals aged between 18 and 60 years old would be recruited based on an OCD diagnose and Yale‐Brown obsessive‐compulsive scale (Y‐BOCS) score ≥16. Those with schizophrenia, substance abuse, developmental disabilities, or severe cognitive dysfunction were excluded, while the patients who had the comorbidity of depression and anxiety symptoms were not excluded from the study. All participants were right‐hand dominant and reported no history of head trauma, neurological disease, or contraindications for MRI. All participants provided written informed consent prior to participation. The study was approved by the Committee on Human Research at the Xinxiang Medical University.

Fifty‐nine eligible patients completed rs‐fMRI scan (19 aged 18–47 received CCT, 19 aged 18–49 received pharmacotherapy plus CCT group (pCCT), and 21 aged 18–55 received pharmacotherapy only (2 participants were over 50 years old). Before treatment, Y‐BOCS symptom checklist and basic demographic data, including age, gender, age at onset of OCD, duration of illness, education, and marriage status, were collected. The Y‐BOCS and fear of negative events were evaluated, and rs‐fMRI was scanned before treatment and after four‐week treatment. The level of fear of negative events (e.g., contamination, losing property) was scaled from 0 (none) to 10 (extreme). The fear of negative events, related to participant's obsession, was evaluated by self‐report globally. The investigators who were responsible for these measurements or rs‐fMRI data analysis were blind to group allocation.

### 2.2 Study design and treatment protocol

**FIGURE 1:**
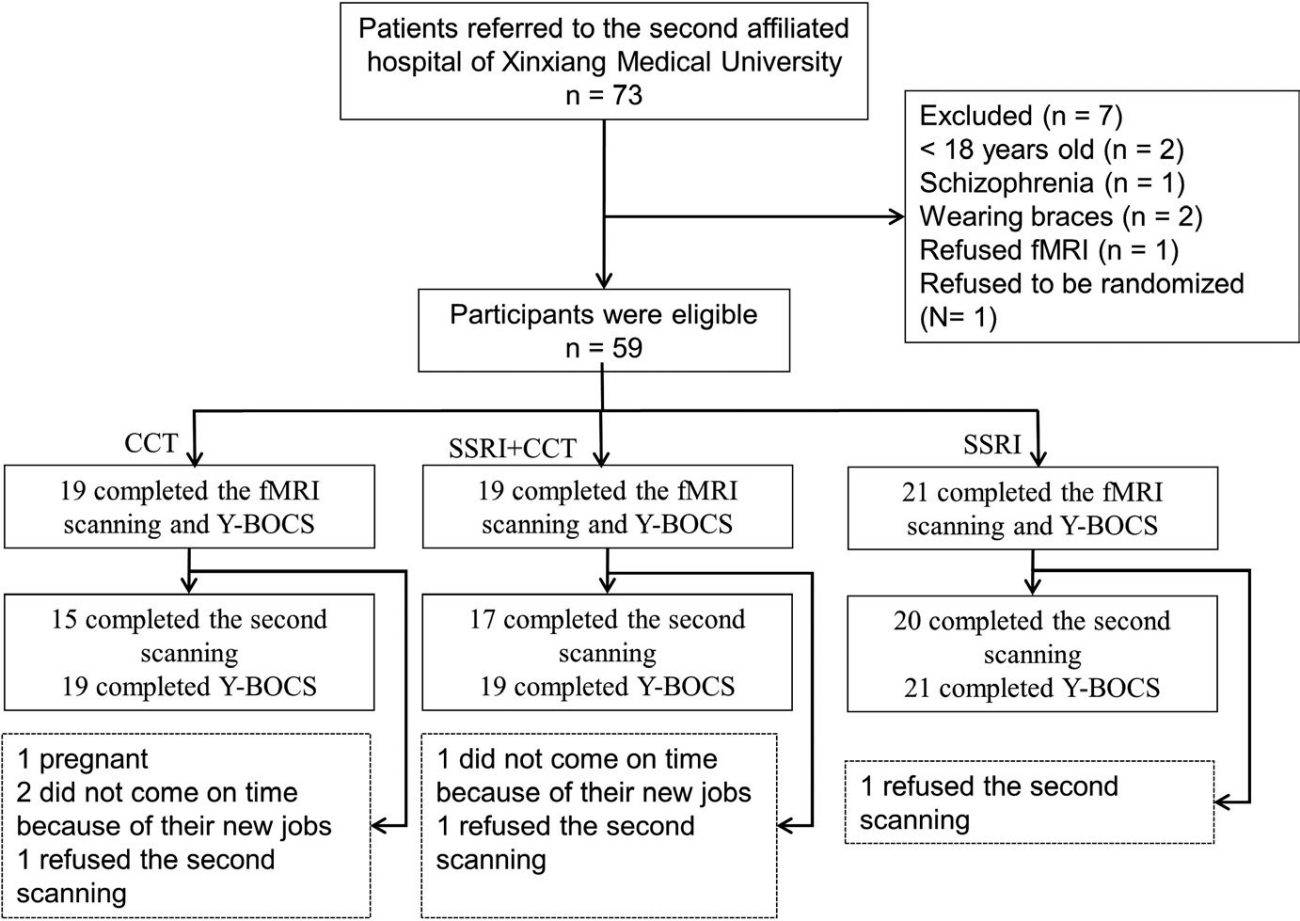
Participant Flow in a Randomized Controlled Trail

The study was designed as a longitudinal study (Figure 1).

#### 2.2.1 Cognitive‐coping therapy (CCT)

Procedure of CCT has been described previously (Hu et al., 2012, 2015; Ma et al., 2013). Briefly, there are four steps in each session of CCT. Step 1 is information collecting and to figure out main symptoms. Step 2 is to identify psychological reason for the symptoms, and it generally attributes to fear/worry of negative events (e.g., handwashing is attributed to a fear of germs or virus or unnamed dirt). Step 3 is to cope with the fear/worry and obsessions. Step 4 is to cope with urge to perform neutralizing behaviors (e.g., sublimation) in order to eliminate over or covert compulsions. Individuals with OCD practiced proper coping strategies at least three times under the guidance of a therapist.

#### 2.2.2 Pharmacotherapy

Before the clinical trials, 16 (76%) patients in pharmacotherapy, and 15 (79%) patients in pCCT took different kinds of SSRI. For those who were medicine naïve, serotonin reuptake inhibitors (SRIs, clomipramine with maximum dose of 225 mg/day) were prescribed to the patients in pharmacotherapy and pCCT groups by the psychiatrists who were blind to CCT assignment. Patients who could not tolerate clomipramine were administered selective serotonin reuptake inhibitors (SSRIs). The dosages of SSRIs would be converted to the dosage of clomipramine (Hayasaka et al., 2015).

### 2.3 The rs‐fMRI scanning and analysis

#### 2.3.1 The rs‐fMRI scanning

All scanning was done on a 3 T MR imaging system (TIM Verio, Siemens, Germany) using a twelve‐channel phased array head coils. After conventional localizer scan and T2 anatomic scan, resting‐state functional images were acquired using an echo‐planar‐imaging (EPI) sequence with the following parameters: 33 axial slices with a slice thickness/gap = 4 mm/0.4mm, repetition time (TR) = 2000 ms, echo time (TE) = 30 ms, flip angle (FA) = 90°, field of view (FOV) = 240 × 240 mm^2^, in‐plan matrix = 64 × 64, resulting in a voxel size of 3.8 × 3.8 × 4 mm^3^, total volumes = 240. For each participant, a high resolution structural T1‐weighted anatomical sequence was scanned in a sagittal orientation using a three‐dimensional magnetization‐prepared rapid gradient‐echo (3D MP‐RAGE) with the following parameters: TR = 1900 ms, TE = 2.52 ms, FA = 15°, slice thickness = 1 mm, data matrix = 256 × 256, isotropic voxel 1 × 1 × 1 mm^3^. To minimize the motion of the subject's head during the study, foam padding was employed. Each patient completed rs‐fMRI scanning before and after 4‐week treatment. During the scanning, participants were instructed simply to remain relaxed with their eyes closed and to think of nothing particularly.

#### 2.3.2 Data preprocessing

The data preprocessing was performed using Pipeline of RESTplus software (Jia et al., 2019). The preprocessing included several steps. First, the first 10 volumes of functional images were discarded to avoid transient signal changes and to let the participant get used to the fMRI scanning environment. Second, slice timing correction and head motion were corrected, and the magnitude of head motion at each time point for 6 parameters (3 for shift and 3 for rotation) was obtained for each subject. The subjects (*n* = 3), who had head motion more than 3.0 mm maximum in any direction and 3°of any angular, were excluded for further analysis. Third, averaged signals from the subject‐specific cerebrospinal fluid (CSF) and white matter (WM) were regressed out to remove the possible spurious variances. Forth, a least squares approach and a 6 parameter spatial transformation were performed, and co‐registration of functional images with 3D‐T1 anatomical images was obtained. Sixth, after spatial normalizing to the standard Montreal Neurological Institute (MNI) template, resampling of the functional images to 3 mm isotropic voxels via parameters of individual 3D image was normalized according to unified segmentation. Finally, the functional images were spatially smoothed with an isotropic Gaussian kernel with a FWHM of 6 mm, and the linear trend within the time series was removed.

#### 2.3.3 ALFF analysis

ALFF analysis was performed using RESTplus software. The procedure has been described previously (Zang et al., 2007). Briefly, after preprocessing, the time series for each voxel was transformed to a frequency domain with a fast Fourier transform (FFT) and the power spectrum was then obtained. Since the power of a given frequency was proportional to the square of the amplitude of this frequency component of the original time series in the time domain, the square root was calculated at each frequency of the power spectrum and the averaged square root was obtained across 0.01 – 0.08 Hz at each voxel. This averaged square root was taken as the ALFF. To standardize the ALFF, the individual maps were divided by their whole‐brain means. The standardized mean ALFF value was used in further statistical analysis.

For the comparison of ALFF before and after treatment, the matched‐sample *t* tests were performed and a contiguity threshold of 228 contiguous voxels (determined by Monte Carlo simulations (Ledberg & Wennberg, 2014)) and voxel‐level *p* < .05 were used as criteria for significant difference corresponding to a corrected *p* < .05 within the whole‐brain mask.

### 2.4 Statistical analysis

ANCOVA with repeated measures and Tukey honest significant difference (HSD) post hoc were performed to test the effects of the treatment, time, and interaction on Y‐BOCS score, using the SPSS 22. Paired *t* tests were performed for the comparison of ALFF value among those before and after controlled OCD patients. A contiguity threshold of 228 contiguous voxels (determined by Monte Carlo simulations (Ledberg & Wennberg, 2014)) and voxel‐level *p* < .05 were used as criteria for significant difference corresponding to a AlphaSim corrected *p* < .05 within the whole‐brain mask. Those were performed using the REST software. Comparisons between Y‐BOCS scores pre‐ and post‐treatment and symptom characteristics were made with unpaired *t* tests. In addition, linear regression analysis was performed to determine the relationship between the changes of ALFF value in cluster (ALFF_post‐treatment_ – ALFF_pretreatment_) and the reduction in percentage of Y‐BOCS score. Also, linear regression was performed to examine the relationship between the change of fear of negative events and severity of symptoms or brain function at rest,

## 3 RESULTS

### 3.1 Demographic and clinical characteristics

For the demographic and clinical characteristics analyses, ANOVA and *t* test were applied in case of continuous variables (e.g., age, education), whereas chi‐square and Fisher's exact tests were applied in case of categorical variables. No significant difference was found among the three groups in age, age at OCD onset, duration of illness, education, Y‐BOCS score, or the distribution of marital status and symptom of OCD. The ratio of male to female was lower in those who accepted pharmacotherapy (Supplemental Table S1). No significant differences in demographic and clinical characteristics, including Y‐BOCS score, were found between remission patients and nonremission patients at baseline (Supplemental Table S2). ANOVA showed that after a four‐week treatment the Y‐BOCS scores in those who received CCT or pCCT were significantly lower than that in those who received pharmacotherapy (*F* = 146.7, *p* < .001). Repeated measure analysis (Wilk's Lambda) showed that the Y‐BOCS scores were significantly lower after the four‐week treatment than pretreatment in CCT group (*F* = 274.1, *p* < .001) and pCCT group (*F* = 285.7, *p* < .001), respectively.

The response rate (Y‐BOCS score was reduced ≥35%) after 4 weeks of treatment was 100% for those received CCT or pCCT. The remission rate (Y‐BOCS score was reduced >80% or less than 8) was around 60%. Both the response rate and the remission rate were not significantly different between CCT and pCCT, but significantly higher than that in patients received pharmacotherapy (*p* < .001) (Table 1). The effect sizes of CCT or pCCT (Cohen's* d* = *M*_1_ − *M*_2_ / s_pooled_, where s_pooled_ = [(s _1_^2^+ s _2_^2^) / 2]^1/2^) were 5.27 or 4.21, respectively.

**TABLE 1:** Comparison of the changes in severity of OCD symptoms (Y‐BOCS score)

	CCT (*n* = 19)	pCCT (*n* = 19)	Pharmacotherapy (*n* = 21)
Pretreatment	26.4 ± 5.1	25.3 ± 6.0	26.0 ± 5.4
Post‐treatment	4.2 ± 5.1	4.7 ± 5.2	24.2 ± 4.1***
Response rate, *N* (%)	19 (100)	19 (100)	1 (4.8)***
Remission rate, *N* (%)	12 (63.1)	11 (57.9)	0 (0.0)***

Note

The response to a treatment was defined as achieving a decline in the Y‐BOCS of ≥35%, and the remission was defined as achieving a reduction in the Y‐BOCS of >80% or less than 8 from the pretreatment. CCT denotes cognitive‐coping therapy; pCCT denotes pharmacotherapy plus cognitive‐coping therapy.

The data were presented as mean ± *SD*.

*** *p* < .001.

### 3.2 Comparison of ALFF value between pre‐ and post‐treatment

**FIGURE 2:**
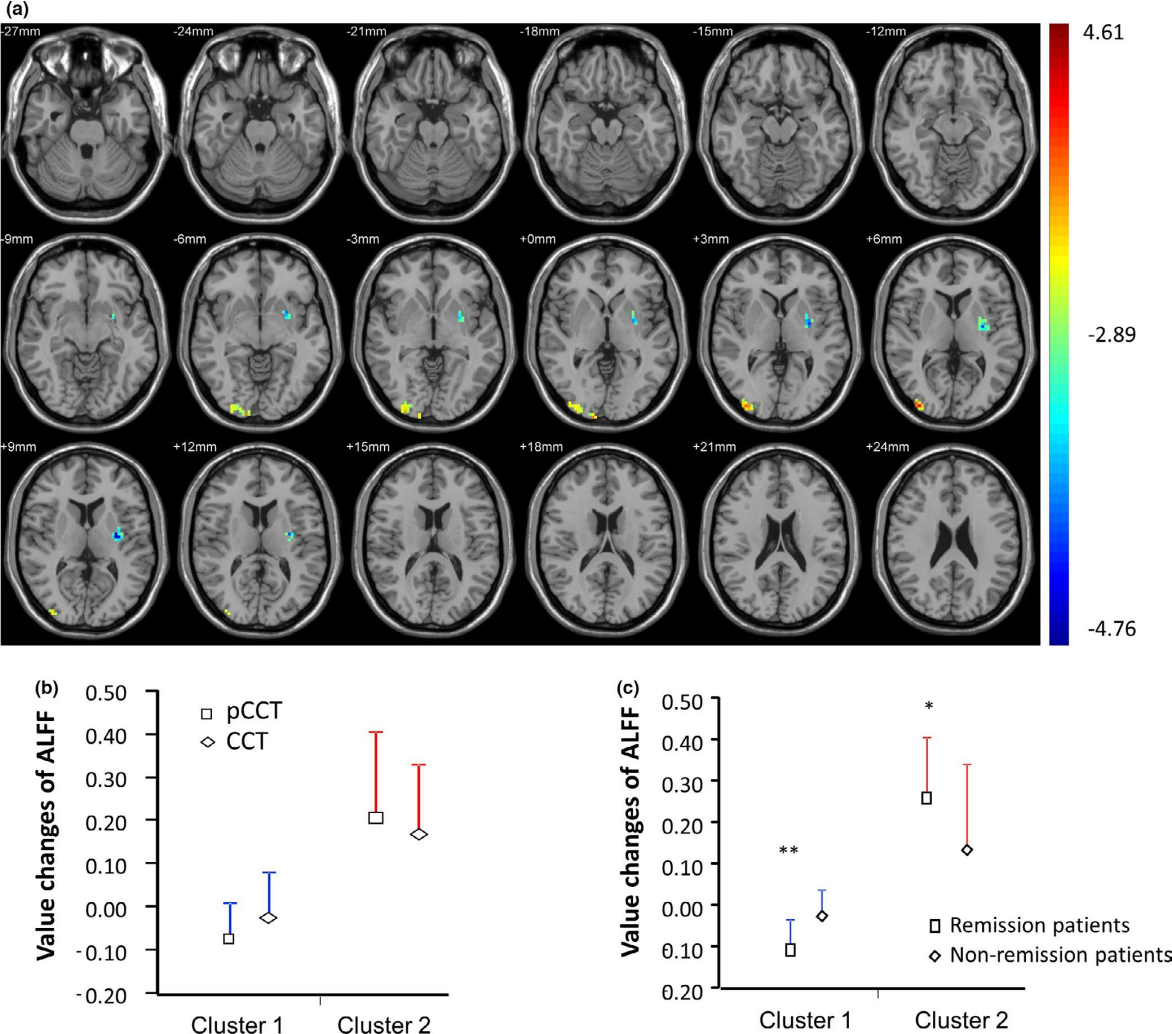
ALFF changes after four weeks treatment in before and after controlled OCD patients. *p < .05; **p < .01. Panel A shows paired *t* tests results the difference of cluster ALFF value (ALFF_post‐treatment_ – ALFF_pretreatment_) among before and after controlled OCD patients. A contiguity threshold of 228 contiguous voxels and voxel‐level *p* < .05 were used as criteria for significant difference corresponding to a corrected *p* < .05 within the whole‐brain mask. After 4‐week treatment, significant changes of ALFF in two clusters were observed. The ALFF in cluster 1 (blue) was significantly decreased, while the ALFF in cluster 2 (red/yellow) was significantly increased. Panel B show no different changes of ALFF in both cluster 1 and cluster 2 between pCCT and CCT. Panel C shows the results compared remission patients with response (nonremission) patients in the two clusters. Mean is presented together with *SD* in the panel b and c

At baseline, no difference in ALFF between the groups of CCT, pCCT, and pharmacotherapy was found. The paired‐sample *t* test (compared post‐treatment with pretreatment) showed two clusters of brain regions with significantly different ALFFs in patients with CCT (combining CCT and pCCT, *n* = 32), since no difference in ALFF was found between the two groups after 4‐week treatment. No cluster was found in patients with pharmacotherapy, compared post‐treatment to baseline (Figure 2a and Supplemental Table S3). The ALFF in cluster 1 (insula, putamen, and postcentral gyrus in left cerebrum) was decreased, while the ALFF in cluster 2 (occipital medial gyrus, occipital inferior gyrus, and lingual gyrus in right hemisphere) was increased (corrected *p* < .05).

The ALFF alterations (ALFF_post‐treatment_ – ALFF_pretreatment_) of the two clusters were not statistically different between the patients treated with CCT and those treated with pCCT (Figure 2b). Therefore, the patients from the two groups were re‐grouped into remission and nonremission. This allowed for the assessment of CCT‐related changes in brain activation and their specificity for successful outcome.(Linden, 2006) The ALFF in cluster 1 was decreased more in remission patients (*n* = 19) than in nonremission patients (*n* = 13) (*p* < .01), while the ALFF in cluster 2 was increased more in remission patients than in nonremission (*p* < .05) (Figure 2c).

### 3.3 Correlation between the alteration of ALFF value and Y‐BOCS score

**FIGURE 3:**
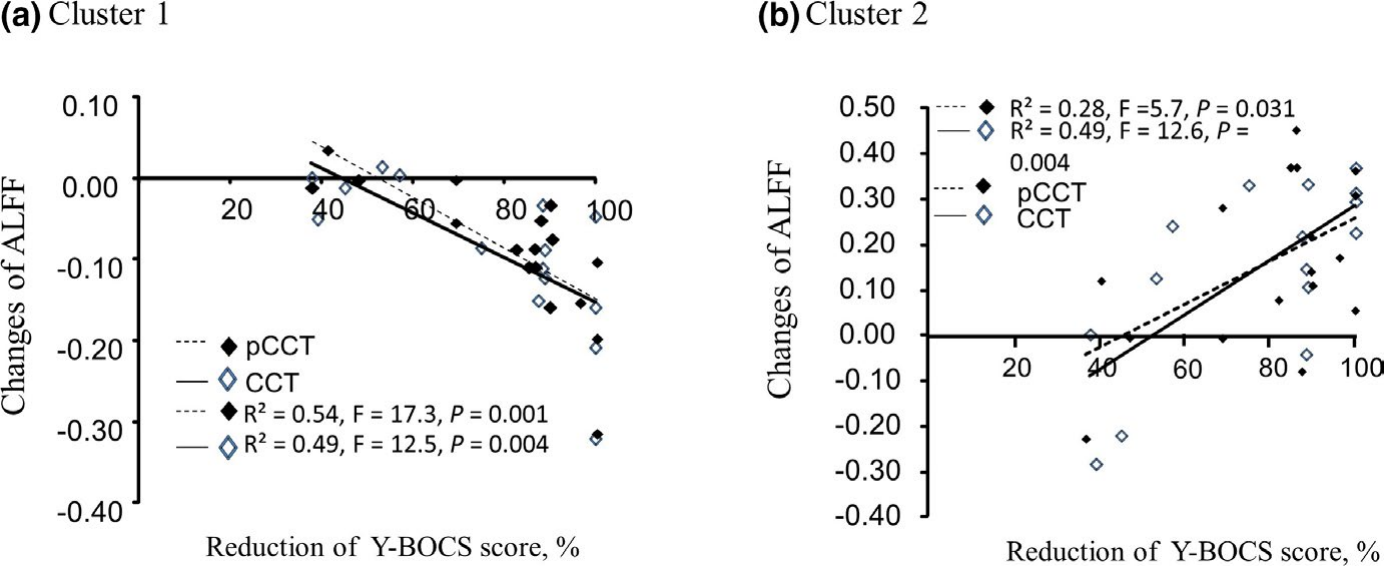
Linear regression analysis between the changes of cluster ALFF and the reduction of Y‐BOCS. Figure 3 shows the relationships between the changes of ALFF (ALFF_post‐treatment_ – ALFF_pretreatment_) and the changes of Y‐BOCS total score after a 4‐week treatment. (a) The linear regression analysis was performed to investigate the relationship between the changes of ALFF in cluster 1 and the changes of Y‐BOCS total score in OCD patients treated with CCT or pCCT, respectively. The beta values were −179.0 (95% CI: −288.1 to −69.8) in CCT group and −173.4 (95% CI: −262.2 to −84.7) in pCCT group. There was no statistical difference in the Beta between groups (*p* > .05). (b) The linear regression analysis was performed to investigate the relationship between the changes of ALFF in cluster 2 and the changes of Y‐BOCS total score in OCD patients treated with CCT or pCCT, respectively. The beta values were 81.7 (95% CI: 57.5 to 82.7) in CCT group and 58.1 (95% CI: 6.1 to 110.0) in pCCT group. There was no statistical difference in the beta between groups (*p* > .05)

The linear regression analysis was performed to investigate the relationship between the difference of pretreatment ALFF value from post‐treatment ALFF value and the Y‐BOCS difference of pre‐ and post‐treated with CCT or pCCT, respectively. The differences of ALFF value in cluster 1 were negatively correlated to the reduction of Y‐BOCS both in CCT group and in pCCT group (*p* < .05, Figure 3a), while the differences of ALFF value in cluster 2 were positively correlated to the reduction of Y‐BOCS (*p* < .05, Figure 3b). Linear regression analysis in combination of the two groups showed similar results, R^2^ equals 0.51 for cluster 1 (*F* = 31.0, *p* < .001) and R^2^ equals 0.38 for cluster 1 (*F* = 18.6, *p* < .001).

### 3.4 Correlation between fear of negative events and Y‐BOCS or the change of ALFF value

**FIGURE 4:**
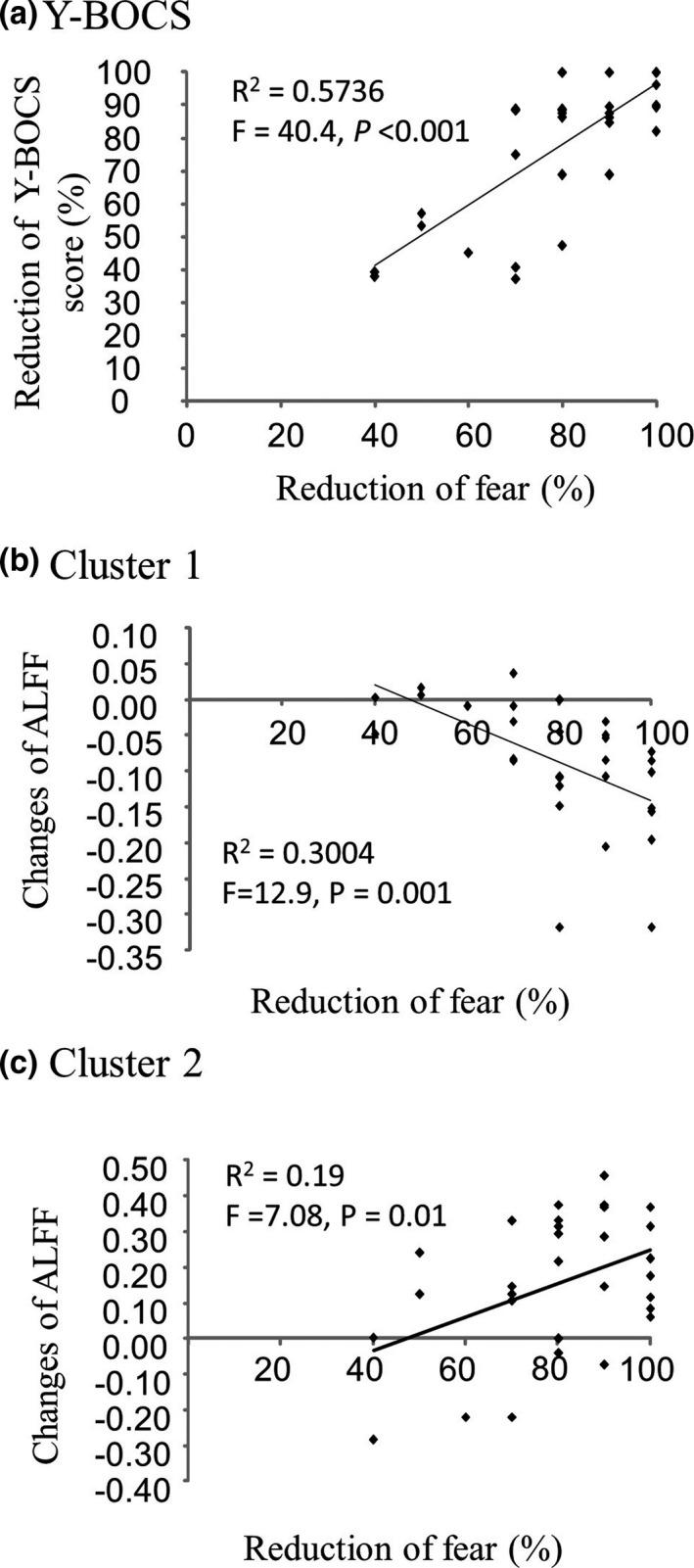
Relationship among intensity of fear, reduction of Y‐BOCS score, and changes of cluster ALFF. Panel a shows that the reduction (%) of fear of negative events was negatively correlated to the reduction of Y‐BOCS score (r = 0.76) after four‐week treatments of CCT and/or pCCT. Panel b shows that the reduction (%) of fear of negative event was negatively correlated to the changes of ALFF (ALFF_post‐treatment_ – ALFF_pretreatment_) in cluster 1 (including insula, putamen, and postcentral gyrus in left cerebrum) (r = 0.55). Panel c shows a positive correlation between the reduction (%) of fear of negative events and the changes of ALFF (ALFF_post‐treatment_ – ALFF_pretreatment_) in cluster 2 (r = 0.62)

Linear regression analysis showed that the reduction of fear of negative events ((pretreatment fear score ‐ post‐treatment fear score)/pretreatment fear score) was positively correlated to the reduction of Y‐BOCS score ((pre–post)/pre) (r = 0.76, *p* < .001; Figure 4a) and the difference of ALFF in cluster 2 (r = 0.62, *p = *.01; Figure 4c), respectively, but negatively correlated to the difference of ALFF in cluster 1 (r = 0.55, *p = *.001; Figure 4b).

## 4 DISCUSSION

Searching for more efficacious treatment for OCD is of great clinical importance because of the partial effectiveness and time‐consumption (O'Neill et al., 2013) of current treatments (Bjorgvinsson et al., 2007). Recently, several studies indicate that CCT for OCD is an effective treatment (Hu et al., 2012; Ma et al., 2013). The efficacy of pCCT is similar in drug‐resistant and nondrug‐resistant adult OCD and is correlated with patient's insight and the disregard of obsessions, but not with the duration of illness, age, gender, and severity of symptoms (Hu et al., 2012; Ma et al., 2013). In this study, the severity of OCD symptoms was significantly decreased through 4‐week treatments of CCT or pCCT and that response rate and remission rate were similar with previously published results. Both response rates and remission rates (above 58%) in participants accepted CCT or pCCT were significantly higher than that in those who received pharmacotherapy only. Furthermore, two clusters of ALFF in brain regions were significantly different in those treated with CCT or pCCT, but no brain region with different ALFF was found in the patients treated with SSRI after four‐week treatment, compared with pretreatment. The alteration of ALFF was not significantly different between CCT and pCCT. These data suggested that the pre‐ to post‐treatment changes in both symptomatic severity and resting state of brain function were primarily related to CCT rather than to pharmacotherapy.

After 4‐week treatment, a significant decreased ALFF value was observed in regions of insula, putamen, and postcentral gyrus located in left cerebrum (cluster 1). The ALFF reduction in this cluster was significantly greater in remission patients than in nonremission patients and was positively correlated to the Y‐BOCS score and fear of negative events at post‐treatments of CCT or pCCT. Also, the reduction in fear of negative event was positively correlated to the reduction in Y‐BOCS. These results suggested that CCT‐induced spontaneous neuronal activity in cluster 1 was associated with decreased fear and Y‐BOCS.

The activation in these areas is involved in fear processing, fear conditioning or extinction (Milad et al., 2013) and in OCD (Apergis‐Schoute et al., 2017). The disorder‐relevant pictures provoke intense OCD symptoms associated with increased activation in the left insula, and neural response toward the disorder‐irrelevant disgusting and fear‐inducing material includes more pronounced insula activation in OCD patients (Schienle et al., 2005). Also, insular cortex is involved in fear acquisition in delay conditioning (Ewald et al., 2014). Strongly symptom‐provoking conditions evoke a dichotomous pattern of deactivation/activation in OCD patients: a deactivation of caudate‐prefrontal circuits accompanied by hyperactivation of putaminal regions (Banca et al., 2015). Increased global brain connect was found in putamen and cerebellar cortex in OCD (Anticevic et al., 2014). Patients with OCD reveal increased BOLD responses in left putamen, bilateral anterior cingulate cortex, and left postcentral gyrus, compared with controls (Remijnse et al., 2013). Our findings demonstrated that the fear of negative events was reduced when the ALFF in these fear‐related regions was decreased after CCT and pCCT and was correlated to the alleviation of OCD symptoms. In addition, D‐cycloserine, a molecule facilitating fear extinction, is demonstrated to improve exposure therapy outcome in patients with OCD (Norberg et al., 2008). These findings suggested the resting‐state activity in these regions, involved in cognitive control (Viard et al., 2005) and cognitively‐induced fear, was associated with OCD.

In the present study, the ALFF value was significantly increased in occipital medial gyrus, occipital inferior gyrus, and lingual gyrus in right hemisphere (cluster 2) after CCT and pCCT treatments. The increased ALFF value in this cluster was positively correlated with the reduction of fear. The ALFF difference between pre‐ and post‐treatment in these regions was greater in remission patients than in nonremission patients, suggesting the increased spontaneous neuronal activity in these regions was associated with the reduction of severity of OCD symptoms. Lingual cortex, a part of occipital lobe, is thinner in OCD patients compared with age‐ and sex‐matched healthy volunteers (Shin et al., 2007). Also, capsulotomy and deep‐brain stimulation lead to similar clinical improvement and increase metabolism in fusiform and lingual gyrus (Suetens et al., 2014). A below‐normal glucose metabolism in the occipital‐parietal area in OCD patients is reported (Nordahl et al., 1989). Goncalves et al hypothesize that fronto‐subcortical activation, consistently found in OCD, may be due to a deactivation of occipital/parietal regions associated with visual‐perceptual processing of incoming social rich stimuli (Goncalves et al., 2010). In this study, the occipital regions were activated in the patients who responded to CCT, although no ALFF alteration in fronto‐subcortical circuit was observed. Our findings suggested that the brain regions (occipital gyrus and lingual gyrus) in cluster of increased ALFF might play a role of regulating or inhibiting the fear‐related region and were involved in the OCD onset and the response to psychotherapy.

In participants who received pharmacotherapy, no cluster of increased or decreased ALFF was found after four‐week treatment compared with pretreatment and that one (4.8%) achieved 35% reduction in Y‐BOCS (response to treatment). A large body of study demonstrates that OCD patients generally do not response to pharmacotherapy in four weeks (Foa, 2010). In a few studies examining how pharmacotherapy affects brain function in OCD patients, the second fMRI scanning is completed after 10 to 16 weeks treatment (Linden, 2006; Shin et al., 2014). Accumulated evidence suggests that the rs‐fMRI is sensitivity to pharmacological challenges in both healthy and clinical conditions. The acute influence of the SSRI citalopram (30 mg) was repeatedly measured in 12 healthy young volunteers with resting‐state fMRI. Citalopram primarily reduces connectivity with the sensorimotor network and structures that are related to self‐referential mechanisms (Klaassens et al., 2017). Bernstein et al report that sertraline has effects on resting‐state functional connectivity in OCD, demonstrating increased striatal connectivity from baseline to 12 weeks compared with controls including the right putamen with the left frontal cortex and insula and the left putamen with the left frontal cortex and pre‐ and postcentral cortices (Bernstein et al., 2019). In the present study, no different cluster based on ALFF was found in patients with pharmacotherapy, which might be related to the fact that 75% of the patients were taking SSRIs when they entered the clinical trial. In addition, the mechanism of pharmacotherapy differs from that of psychotherapy (e.g., CBT) (Ressler & Rothbaum, 2013). In this study, baseline imaging measures are obtained before the treatments of either CCT, pCCT, or pharmacotherapy, after which patients are classified as remission or nonremission based on change of Y‐BOCS. This method allows for the assessment of CCT‐related changes in brain activation and their specificity for successful outcome (Linden, 2006).

We did not find the therapeutic impact on the orbitofrontal cortex, a region of widely reported cortico‐striatal‐thalamocortical circuit involved in OCD. A previous study demonstrates increased ALFF in the bilateral orbitofrontal cortex and anterior cingulate cortex, as well as decreased ALFF in the bilateral cerebellum and parietal cortex (Hou et al., 2012), while another study reports decreased ALFF in some areas including right orbitofrontal and left parietal in OCD patients (Zhao et al., 2017). Both studies are compared with healthy controls. Possible explanations for the discrepancies include differences in sample characteristics (e.g., various symptoms) and a broad range of brain regions involved in OCD (Heinzel et al., 2018). The heterogeneity‐related factors such as severity of illness and interventions should be carefully controlled in future studies.

Several limitations of our study need to be noted. First, the sample size was relatively small, especially for the rs‐fMRI, which limited our ability to do further analysis across groups. Second, the ALFF, which is used in this study, has been previously indicated to be sensitive to physiological noises such as respiration, cardiac action, and motion (Zou et al., 2008). Potential sensitivity was reported in the cistern areas of the brain as well as potentially in the limbic and occipital‐temporal areas. Fractional ALFF (fALFF) was proposed to suppress nonspecific signal components in these areas and to improve the results obtained in this study. Although the ALFF is recently reported to have higher discriminative power for OCD than the fALFF (Bu et al., 2019), the potential effects of those physiological functions on the neuroimaging should be taken into account in future studies. Third, OCD patients who had comorbidity with depression or anxiety were not excluded, as the comorbidity issue is common in the clinically psychiatric diagnosis. Some evidence suggest that OCD patients with comorbidity of depression or anxiety have different neuroimaging results, compared with those without (Cardoner et al., 2007; Kong et al., 2020; Remijnse et al., 2013; Saxena et al., 2001; Tadayonnejad et al., 2018; Thorsen et al., 2018). Our data warranted larger sample size in future studies is needed to examine the effect of CCT on OCD patients with the comorbidity and those without.

In summary, the present study provided evidence that an increased ALFF cluster in the right cerebrum and a decreased ALFF cluster in the left cerebrum were associated with the response to CCT for OCD. Up to 60% of OCD patients achieved a remission with short‐term CCT, and the efficacy was related to the alteration of the resting‐state brain function in the two clusters that mainly involve fear processing, conditioning or extinction, and cognitively‐induced fear. Our finding suggested that the effectiveness of CCT was related to the alteration of resting‐state brain function—the brain plasticity.

## CONFLICT OF INTERESTS

All authors declare that they have no conflict of interest.

## AUTHOR CONTRIBUTIONS

Jian‐Dong Ma, and Xian‐Zhang Hu contributed to study concept and design. Jian‐Dong Ma, Chang‐Hong Wang, Ping Huang, Heng‐Fen Li, Shao‐Jie Kou, Zhi‐Rong Li, Hong‐Kai Lian, and Xian‐Zhang Hu were responsible for diagnosis and clinical data collection. De‐En Sang, Hong‐Zeng Zhao, and Xian‐Zhang Hu collected the rs‐fMRI data and performed ALFF analysis. Xunan Wang, Li‐Jing Shi, and Xian‐Zhang Hu data established dataset. Jian‐Dong Ma and Xian‐Zhang Hu analyzed and interpreted data. Jian‐Dong Ma drafted the manuscript. Chang‐Hong Wang, Ping Huang, Heng‐Fen Li, Shao‐Jie Kou, Zhi‐Rong Li, Hong‐Kai Lian, and Xian‐Zhang Hu critically revised the manuscript for important intellectual content. Xian‐Zhang Hu obtained funding.

## Supporting information

Supplementary Material

## Data Availability

Research data are not shared.

## References

[brb32059-bib-0001] Anticevic, A. , Hu, S. , Zhang, S. , Savic, A. , Billingslea, E. , Wasylink, S. , Repovs, G. , Cole, M. W. , Bednarski, S. , Krystal, J. H. , Bloch, M. H. , Li, C.‐S. , & Pittenger, C. (2014). Global resting‐state functional magnetic resonance imaging analysis identifies frontal cortex, striatal, and cerebellar dysconnectivity in obsessive‐compulsive disorder. Biological Psychiatry, 75(8), 595–605. 10.1016/j.biopsych.2013.10.021 24314349 PMC3969771

[brb32059-bib-0002] Apergis‐Schoute, A. M. , Gillan, C. M. , Fineberg, N. A. , Fernandez‐Egea, E. , Sahakian, B. J. , & Robbins, T. W. (2017). Neural basis of impaired safety signaling in Obsessive Compulsive Disorder. Proceedings of the National Academy of Sciences of the United States of America, 114(12), 3216–3221. 10.1073/pnas.1609194114 28265059 PMC5373407

[brb32059-bib-0003] Banca, P. , Voon, V. , Vestergaard, M. D. , Philipiak, G. , Almeida, I. , Pocinho, F. , Relvas, J. , & Castelo‐Branco, M. (2015). Imbalance in habitual versus goal directed neural systems during symptom provocation in obsessive‐compulsive disorder. Brain, 138(Pt 3), 798–811. 10.1093/brain/awu379 25567322 PMC4339772

[brb32059-bib-0004] Baxter, L. R. Jr , Schwartz, J. M. , Bergman, K. S. , Szuba, M. P. , Guze, B. H. , Mazziotta, J. C. (1992). Caudate glucose metabolic rate changes with both drug and behavior therapy for obsessive‐compulsive disorder. Archives of General Psychiatry, 49(9), 681–689. 10.1001/archpsyc.1992.01820090009002 1514872

[brb32059-bib-0005] Bernstein, G. A. , Cullen, K. R. , Harris, E. C. , Conelea, C. A. , Zagoloff, A. D. , Carstedt, P. A. , Lee, S. S. , & Mueller, B. A. (2019). Sertraline effects on striatal resting‐state functional connectivity in youth with obsessive‐compulsive disorder: A pilot study. Journal of the American Academy of Child and Adolescent Psychiatry, 58(5), 486–495. 10.1016/j.jaac.2018.07.897 30768407 PMC6487209

[brb32059-bib-0006] Bjorgvinsson, T. , Hart, J. , & Heffelfinger, S. (2007). Obsessive‐compulsive disorder: Update on assessment and treatment. Journal of Psychiatric Practice, 13(6), 362–372. 10.1097/01.pra.0000300122.76322.ad 18032981

[brb32059-bib-0007] Bu, X. , Hu, X. , Zhang, L. , Li, B. , Zhou, M. , Lu, L. U. , Hu, X. , Li, H. , Yang, Y. , Tang, W. , Gong, Q. , & Huang, X. (2019). Investigating the predictive value of different resting‐state functional MRI parameters in obsessive‐compulsive disorder. Translational Psychiatry, 9(1), 17. 10.1038/s41398-018-0362-9 30655506 PMC6336781

[brb32059-bib-0008] Cardoner, N. , Soriano‐Mas, C. , Pujol, J. , Alonso, P. , Harrison, B. J. , Deus, J. , Hernández‐Ribas, R. , Menchón, J. M. , & Vallejo, J. (2007). Brain structural correlates of depressive comorbidity in obsessive‐compulsive disorder. NeuroImage, 38(3), 413–421. 10.1016/j.neuroimage.2007.07.039 17889563

[brb32059-bib-0009] Ewald, H. , Glotzbach‐Schoon, E. , Gerdes, A. B. , Andreatta, M. , Muller, M. , Muhlberger, A. , & Pauli, P. (2014). Delay and trace fear conditioning in a complex virtual learning environment‐neural substrates of extinction. Frontiers in Human Neuroscience, 8, 323. 10.3389/fnhum.2014.00323 24904363 PMC4034409

[brb32059-bib-0010] Foa, E. B. (2010). Cognitive behavioral therapy of obsessive‐compulsive disorder. Dialogues in Clinical Neuroscience, 12(2), 199–207. 20623924 10.31887/DCNS.2010.12.2/efoa PMC3181959

[brb32059-bib-0011] Foa, E. B. , Simpson, H. B. , Rosenfield, D. , Liebowitz, M. R. , Cahill, S. P. , Huppert, J. D. , Bender Jr, J. , McLean, C. P. , Maher, M. J. , Campeas, R. , Hahn, C.‐G. , Imms, P. , Pinto, A. , Powers, M. B. , Rodriguez, C. I. , Van Meter, P. E. , Vermes, D. , & Williams, M. T. (2015). Six‐month outcomes from a randomized trial augmenting serotonin reuptake inhibitors with exposure and response prevention or risperidone in adults with obsessive‐compulsive disorder. Journal of Clinical Psychiatry, 76(4), 440–446. 10.4088/JCP.14m09044 PMC4524354 25375780

[brb32059-bib-0012] Goncalves, O. F. , Marques, T. R. , Lori, N. F. , Sampaio, A. , & Branco, M. C. (2010). Obsessive‐compulsive disorder as a visual processing impairment. Medical Hypotheses, 74(1), 107–109. 10.1016/j.mehy.2009.07.048 19695786

[brb32059-bib-0013] Hayasaka, Y. U. , Purgato, M. , Magni, L. R. , Ogawa, Y. , Takeshima, N. , Cipriani, A. , Barbui, C. , Leucht, S. , & Furukawa, T. A. (2015). Dose equivalents of antidepressants: Evidence‐based recommendations from randomized controlled trials. Journal of Affective Disorders, 180, 179–184. 10.1016/j.jad.2015.03.021 25911132

[brb32059-bib-0014] Heinzel, S. , Kaufmann, C. , Grützmann, R. , Hummel, R. , Klawohn, J. , Riesel, A. , Bey, K. , Lennertz, L. , Wagner, M. , & Kathmann, N. (2018). Neural correlates of working memory deficits and associations to response inhibition in obsessive compulsive disorder. NeuroImage: Clinical, 17, 426–434. 10.1016/j.nicl.2017.10.039 29159055 PMC5683807

[brb32059-bib-0015] Holes, R. E. , Yudofsky, S. C. , & Roberts, L. W. (2014). Textbook of Psychiatry. The American Psychiatric Publishing.

[brb32059-bib-0016] Hollander, E. , Allen, A. , Steiner, M. , Wheadon, D. E. , Oakes, R. , & Burnham, D. B. ; Paroxetine OCD Study Group (2003). Acute and long‐term treatment and prevention of relapse of obsessive‐compulsive disorder with paroxetine. Journal of Clinical Psychiatry, 64(9), 1113–1121. 10.4088/jcp.v64n0919 14628989

[brb32059-bib-0017] Hollander, E. , Bienstock, C. A. , Koran, L. M. , Pallanti, S. , Marazziti, D. , Rasmussen, S. A. , & Zohar, J. (2002). Refractory obsessive‐compulsive disorder: State‐of‐the‐art treatment. Journal of Clinical Psychiatry, 63(Suppl 6), 20–29. 12027116

[brb32059-bib-0018] Hou, J. , Wu, W. , Lin, Y. , Wang, J. , Zhou, D. , Guo, J. , Gu, S. , He, M. , Ahmed, S. , Hu, J. , Qu, W. , & Li, H. (2012). Localization of cerebral functional deficits in patients with obsessive‐compulsive disorder: A resting‐state fMRI study. Journal of Affective Disorders, 138(3), 313–321. 10.1016/j.jad.2012.01.022 22331021

[brb32059-bib-0019] Hu, X.‐Z. (2010). A novel cognitive‐coping therapy for obsessive‐complusive disorder. Journal of Applied Clinical Pediatrics, 25(24), 1848–1851.

[brb32059-bib-0020] Hu, X.‐Z. , & Ma, J.‐D. (2011). Clinical effect of pharmacotherapy combined with cognitive‐coping therapy on obsessive‐compulsive disorder. Journal of Xinxiang Medical College, 28(1), 68–72.

[brb32059-bib-0021] Hu, X.‐Z. , Ma, J.‐D. , Huang, P. , Shan, X.‐W. , Zhang, Z.‐H. , Zhang, J.‐H. , Ouyang, H. , Kou, S.‐J. , Li, Z.‐R. , Wang, S.‐F. , Zhao, H.‐Z. , Wang, H. , & Wang, C.‐H. (2015). Highly efficacious cognitive‐coping therapy for overt or covert compulsions. Psychiatry Research, 229(3), 732–738. 10.1016/j.psychres.2015.08.010 26275705

[brb32059-bib-0022] Hu, X. Z. , Wen, Y. S. , Ma, J. D. , Han, D. M. , Li, Y. X. , & Wang, S. F. (2012). A promising randomized trial of a new therapy for obsessive‐compulsive disorder. Brain and Behavior, 2(4), 443–454. 10.1002/brb3.67 22950048 PMC3432967

[brb32059-bib-0023] Huang, Y. , Wang, Y. U. , Wang, H. , Liu, Z. , Yu, X. , Yan, J. , Yu, Y. , Kou, C. , Xu, X. , Lu, J. , Wang, Z. , He, S. , Xu, Y. , He, Y. , Li, T. , Guo, W. , Tian, H. , Xu, G. , Xu, X. , … Wu, Y. (2019). Prevalence of mental disorders in China: A cross‐sectional epidemiological study. Lancet Psychiatry, 6(3), 211–224. 10.1016/S2215-0366(18)30511-X 30792114

[brb32059-bib-0024] Jia, X. , Wang, J. , Sun, H. , Zhang, H. , Liao, W. , Wang, Z. , & Zang, Y. (2019). RESTplus: An improved toolkit for resting‐state functional magnetic resonance imaging data processing. Chinese Science Bulletin, 64, 953–954. 10.1016/j.scib.2019.05.008 36659803

[brb32059-bib-0025] Klaassens, B. L. , Rombouts, S. A. R. B. , Winkler, A. M. , van Gorsel, H. C. , van der Grond, J. , & van Gerven, J. M. A. (2017). Time related effects on functional brain connectivity after serotonergic and cholinergic neuromodulation. Human Brain Mapping, 38(1), 308–325. 10.1002/hbm.23362 27622387 PMC5215384

[brb32059-bib-0026] Kong, X.‐Z. , Boedhoe, P. S. W. , Abe, Y. , Alonso, P. , Ameis, S. H. , Arnold, P. D. , Assogna, F. , Baker, J. T. , Batistuzzo, M. C. , Benedetti, F. , Beucke, J. C. , Bollettini, I. , Bose, A. , Brem, S. , Brennan, B. P. , Buitelaar, J. , Calvo, R. , Cheng, Y. , Cho, K. I. K. , … Francks, C. (2020). Mapping cortical and subcortical asymmetry in obsessive‐compulsive disorder: Findings from the ENIGMA Consortium. Biological Psychiatry, 87(12), 1022–1034. 10.1016/j.biopsych.2019.04.022 31178097 PMC7094802

[brb32059-bib-0027] Koran, L. M. , Hackett, E. , Rubin, A. , Wolkow, R. , & Robinson, D. (2002). Efficacy of sertraline in the long‐term treatment of obsessive‐compulsive disorder. American Journal of Psychiatry, 159(1), 88–95. 10.1176/appi.ajp.159.1.88 11772695

[brb32059-bib-0028] Krebs, G. , Isomura, K. , Lang, K. , Jassi, A. , Heyman, I. , Diamond, H. , Advani, J. , Turner, C. , & Mataix‐Cols, D. (2015). How resistant is 'treatment‐resistant' obsessive‐compulsive disorder in youth? British Journal of Clinical Psychology, 54(1), 63–75. 10.1111/bjc.12061 25130442

[brb32059-bib-0029] Külz, A. K. , Landmann, S. , Cludius, B. , Hottenrott, B. , Rose, N. , Heidenreich, T. , Hertenstein, E. , Voderholzer, U. , & Moritz, S. (2014). Mindfulness‐based cognitive therapy in obsessive‐compulsive disorder: Protocol of a randomized controlled trial. BMC Psychiatry, 14(1), 314. 10.1186/s12888-014-0314-8 25403813 PMC4239327

[brb32059-bib-0030] Ledberg, A. , & Wennberg, P. (2014). Estimating the size of hidden populations from register data. BMC Medical Research Methodology, 14, 58. 10.1186/1471-2288-14-58 24766871 PMC4011782

[brb32059-bib-0031] Lee, M. H. , Smyser, C. D. , & Shimony, J. S. (2013). Resting‐state fMRI: A review of methods and clinical applications. American Journal of Neuroradiology, 34(10), 1866–1872. 10.3174/ajnr.A3263 22936095 PMC4035703

[brb32059-bib-0032] Linden, D. E. (2006). How psychotherapy changes the brain–the contribution of functional neuroimaging. Molecular Psychiatry, 11(6), 528–538. 10.1038/sj.mp.4001816 16520823

[brb32059-bib-0033] Ma, J.‐D. , Wang, C.‐H. , Li, H.‐F. , Zhang, X.‐L. , Zhang, Y.‐L. , Hou, Y.‐H. , Liu, X.‐H. , & Hu, X.‐Z. (2013). Cognitive‐coping therapy for obsessive‐compulsive disorder: A randomized controlled trial. Journal of Psychiatric Research, 47(11), 1785–1790. 10.1016/j.jpsychires.2013.08.002 23988179

[brb32059-bib-0034] Math, S. B. , & Janardhan Reddy, Y. C. (2007). Issues in the pharmacological treatment of obsessive‐compulsive disorder. International Journal of Clinical Practice, 61(7), 1188–1197. 10.1111/j.1742-1241.2007.01356.x 17511795

[brb32059-bib-0035] Milad, M. R. , Furtak, S. C. , Greenberg, J. L. , Keshaviah, A. , Im, J. J. , Falkenstein, M. J. , Jenike, M. , Rauch, S. L. , & Wilhelm, S. (2013). Deficits in conditioned fear extinction in obsessive‐compulsive disorder and neurobiological changes in the fear circuit. JAMA Psychiatry, 70(6), 608–618. 10.1001/jamapsychiatry.2013.914 23740049

[brb32059-bib-0036] Norberg, M. M. , Krystal, J. H. , & Tolin, D. F. (2008). A meta‐analysis of D‐cycloserine and the facilitation of fear extinction and exposure therapy. Biological Psychiatry, 63(12), 1118–1126. 10.1016/j.biopsych.2008.01.012 18313643

[brb32059-bib-0037] Nordahl, T. E. , Benkelfat, C. , Semple, W. E. , Gross, M. , King, A. C. , & Cohen, R. M. (1989). Cerebral glucose metabolic rates in obsessive compulsive disorder. Neuropsychopharmacology, 2(1), 23–28. 10.1016/0893-133X(89)90003-1 2803479

[brb32059-bib-0038] O'Neill, J. , Gorbis, E. , Feusner, J. D. , Yip, J. C. , Chang, S. , Maidment, K. M. , Levitt, J. G. , Salamon, N. , Ringman, J. M. , & Saxena, S. (2013). Effects of intensive cognitive‐behavioral therapy on cingulate neurochemistry in obsessive‐compulsive disorder. Journal of Psychiatric Research, 47(4), 494–504. 10.1016/j.jpsychires.2012.11.010 23290560 PMC3672238

[brb32059-bib-0039] Reggente, N. , Moody, T. D. , Morfini, F. , Sheen, C. , Rissman, J. , O'Neill, J. , & Feusner, J. D. (2018). Multivariate resting‐state functional connectivity predicts response to cognitive behavioral therapy in obsessive‐compulsive disorder. Proceedings of the National Academy of Sciences of the United States of America, 115(9), 2222–2227. 10.1073/pnas.1716686115 29440404 PMC5834692

[brb32059-bib-0040] Remijnse, P. L. , van den Heuvel, O. A. , Nielen, M. M. A. , Vriend, C. , Hendriks, G.‐J. , Hoogendijk, W. J. G. , Uylings, H. B. M. , & Veltman, D. J. (2013). Cognitive inflexibility in obsessive‐compulsive disorder and major depression is associated with distinct neural correlates. PLoS One, 8(4). 10.1371/journal.pone.0059600 PMC3634812 23637737

[brb32059-bib-0041] Ressler, K. J. , & Rothbaum, B. O. (2013). Augmenting obsessive‐compulsive disorder treatment: From brain to mind. JAMA Psychiatry, 70(11), 1129–1131. 10.1001/jamapsychiatry.2013.2116 24026506

[brb32059-bib-0042] Rufer, M. , Hand, I. , Alsleben, H. , Braatz, A. , Ortmann, J. , Katenkamp, B. , Fricke, S. , & Peter, H. (2005). Long‐term course and outcome of obsessive‐compulsive patients after cognitive‐behavioral therapy in combination with either fluvoxamine or placebo: A 7‐year follow‐up of a randomized double‐blind trial. European Archives of Psychiatry and Clinical Neuroscience, 255(2), 121–128. 10.1007/s00406-004-0544-8 15812606

[brb32059-bib-0043] Sang, D. E. , Shi, L. J. , Yue, K. C. , He, C. Y. , Zhao, H. Z. , Wang, C. H. , & Hu, X. Z. (2020). Clinical remission of a treatment‐refractory individual with severe repetitive rituals and rumination. Asian Journal of Psychiatry, 47, 101878. 10.1016/j.ajp.2019.101878 31756555

[brb32059-bib-0044] Saxena, S. , Brody, A. L. , Ho, M. L. , Alborzian, S. , Ho, M. K. , Maidment, K. M. , Huang, S.‐C. , Wu, H.‐M. , Au, S. C. , & Baxter, L. R. (2001). Cerebral metabolism in major depression and obsessive‐compulsive disorder occurring separately and concurrently. Biological Psychiatry, 50(3), 159–170. 10.1016/S0006-3223(01)01123-4 11513814

[brb32059-bib-0045] Saxena, S. , Gorbis, E. , O'Neill, J. , Baker, S. K. , Mandelkern, M. A. , Maidment, K. M. , Chang, S. , Salamon, N. , Brody, A. L. , Schwartz, J. M. , & London, E. D. (2009). Rapid effects of brief intensive cognitive‐behavioral therapy on brain glucose metabolism in obsessive‐compulsive disorder. Molecular Psychiatry, 14(2), 197–205. 10.1038/sj.mp.4002134 18180761 PMC2893580

[brb32059-bib-0046] Schienle, A. , Schafer, A. , Stark, R. , Walter, B. , & Vaitl, D. (2005). Neural responses of OCD patients towards disorder‐relevant, generally disgust‐inducing and fear‐inducing pictures. International Journal of Psychophysiology, 57(1), 69–77. 10.1016/j.ijpsycho.2004.12.013 15935263

[brb32059-bib-0047] Shin, D.‐J. , Jung, W. H. , He, Y. , Wang, J. , Shim, G. , Byun, M. S. , Jang, J. H. , Kim, S. N. , Lee, T. Y. , Park, H. Y. , & Kwon, J. S. (2014). The effects of pharmacological treatment on functional brain connectome in obsessive‐compulsive disorder. Biological Psychiatry, 75(8), 606–614. 10.1016/j.biopsych.2013.09.002 24099506

[brb32059-bib-0048] Shin, Y.‐W. , Yoo, S. Y. , Lee, J. K. , Ha, T. H. , Lee, K. J. , Lee, J. M. , Kim, I. Y. , Kim, S. I. , & Kwon, J. S. (2007). Cortical thinning in obsessive compulsive disorder. Human Brain Mapping, 28(11), 1128–1135. 10.1002/hbm.20338 17525985 PMC6871365

[brb32059-bib-0049] Simpson, H. B. , Franklin, M. E. , Cheng, J. , Foa, E. B. , & Liebowitz, M. R. (2005). Standard criteria for relapse are needed in obsessive‐compulsive disorder. Depression and Anxiety, 21(1), 1–8. 10.1002/da.20052 15806597

[brb32059-bib-0050] Stern, E. R. , Fitzgerald, K. D. , Welsh, R. C. , Abelson, J. L. , & Taylor, S. F. (2012). Resting‐state functional connectivity between fronto‐parietal and default mode networks in obsessive‐compulsive disorder. PLoS One, 7(5), 3. 10.1371/journal.pone.0036356 PMC3343054 22570705

[brb32059-bib-0051] Suetens, K. , Nuttin, B. , Gabriels, L. , & Van Laere, K. (2014). Differences in metabolic network modulation between capsulotomy and deep‐brain stimulation for refractory obsessive‐compulsive disorder. Journal of Nuclear Medicine, 55(6), 951–959. 10.2967/jnumed.113.126409 24722531

[brb32059-bib-0052] Tadayonnejad, R. , Deshpande, R. , Ajilore, O. , Moody, T. , Morfini, F. , Ly, R. , O'Neill, J. , & Feusner, J. D. (2018). Pregenual anterior cingulate dysfunction associated with depression in OCD: An integrated multimodal fMRI/(1)H MRS study. Neuropsychopharmacology, 43(5), 1146–1155. 10.1038/npp.2017.249 29052616 PMC5854805

[brb32059-bib-0053] Thorsen, A. L. , Hagland, P. , Radua, J. , Mataix‐Cols, D. , Kvale, G. , Hansen, B. , & van den Heuvel, O. A. (2018). Emotional processing in obsessive‐compulsive disorder: A systematic review and meta‐analysis of 25 functional neuroimaging studies. Biological Psychiatry: Cognitive Neuroscience and Neuroimaging, 3(6), 563–571. 10.1016/j.bpsc.2018.01.009 29550459 PMC5994188

[brb32059-bib-0054] Viard, A. , Flament, M. F. , Artiges, E. , Dehaene, S. , Naccache, L. , Cohen, D. , Mazet, P. , Mouren, M.‐C. , & Martinot, J.‐L. (2005). Cognitive control in childhood‐onset obsessive‐compulsive disorder: A functional MRI study. Psychological Medicine, 35(7), 1007–1017. 10.1017/S0033291704004295 16045067

[brb32059-bib-0055] Yu‐Feng, Z. , Yong, H. E. , Chao‐Zhe, Z. , Qing‐Jiu, C. , Man‐Qiu, S. , Meng, L. , Li‐Xia, T. , Tian‐Zi, J. , & Yu‐Feng, W. (2007). Altered baseline brain activity in children with ADHD revealed by resting‐state functional MRI. Brain Dev, 29(2), 83–91. 10.1016/j.braindev.2006.07.002 16919409

[brb32059-bib-0056] Zhao, H.‐Z. , Wang, C.‐H. , Gao, Z.‐Z. , Ma, J.‐D. , Huang, P. , Li, H.‐F. , Sang, D.‐E. , Shan, X.‐W. , Kou, S.‐J. , Li, Z.‐R. , Ma, L. I. , Zhang, Z.‐H. , Zhang, J.‐H. , Ouyang, H. , Lian, H.‐K. , Zang, Y.‐F. , & Hu, X.‐Z. (2017). Effectiveness of cognitive‐coping therapy and alteration of resting‐state brain function in obsessive‐compulsive disorder. Journal of Affective Disorders, 208, 184–190. 10.1016/j.jad.2016.10.015 27792961

[brb32059-bib-0057] Zou, Q.‐H. , Zhu, C.‐Z. , Yang, Y. , Zuo, X.‐N. , Long, X.‐Y. , Cao, Q.‐J. , Wang, Y.‐F. , & Zang, Y.‐F. (2008). An improved approach to detection of amplitude of low‐frequency fluctuation (ALFF) for resting‐state fMRI: Fractional ALFF. Journal of Neuroscience Methods, 172(1), 137–141. 10.1016/j.jneumeth.2008.04.012 18501969 PMC3902859

