# Supplementary material for: Effects of short‐term cognitive‐coping therapy on resting‐state brain function in obsessive‐compulsive disorder
Source: Brain Behav. 2021 Feb 9;11(4):e02059. doi: 10.1002/brb3.2059 (PMC8035441; doi:10.1002/brb3.2059)
Supplement: Supplementary file 1 — Supplementary Material [file BRB3-11-e02059-s001.doc]

**Supplemental Material**

**Methods**

**Cognitive-coping therapy**

Cognitive-coping therapy (CCT) for OCD is developed based on the cognitive theory and stress-coping theory . Coping is defined constantly changing cognitive and behavioral efforts to manage specific external and/or internal demands that are appraised as taxing” or “exceeding the resources of the person . Common coping strategies include three subtypes: problem-focused coping (information seeking and problem solving), emotion-focused coping (expressing emotion and regulating emotions) , and appraisal focused coping (denial, acceptance, social comparison, redefinition, and logical analysis) .

In clinical practice since 1996, Dr. Hu reputed that OCD basically has four main components: obsessions, compulsions, anxiety, and fear of negative events, which may be considered to be stressors and could be coped with using proper coping strategies. It is observed that the anxious level will decrease after cognitive restructuring focusing on the fear and that the severity of the symptoms can be apparently reduced rapidly when appropriate coping strategies are used correctly.

It should be noted that the outcome of CCT has to do with its enhanced cognitive therapy. The cognitive therapy focuses on the *fear* of negative events firstly*,* obsessions secondly, and then compulsions. The goal of cognitive therapy is to change the patients’ attitude and the way of thinking related to their symptoms, especially the fear of negative events. In that way, the patients are thought to utilize the appraisal-focused coping strategies, because appraisal-focused strategies occur when individuals modify the way they think by altering goals and values . CCT emphasize that it is the fear of negative events rather than the obsessions or obsession-related anxiety leads to a motivation to perform the compulsions . The compulsions will not be necessary when the fear of negative events, obsessions, and the motivation to perform the compulsions are properly coped with.

CCT has several characters in aspects of treatment that differ from other psychotherapies. First, CCT takes the fear as a treatment target. Second, CCT involves coping strategies. Third, CCT encourages patients not “talk back” to OCD, but untangle with obsessions or compulsions. Finally, CCT does not use exposure and response prevention (ERP).

There are four steps in each session of CCT. The therapeutic steps are listed below.

Step 1 was information collecting.

Step 2 was to identify fear/worry of negative events and to cope with the fear/worry (e.g. rationale). When OCD patients asked why the behavioral or mental rituals were performed, their answers were usually related to a fear/worry of negative events directly or indirectly. The roles of FEAR/WORRY in the onset of OCD were then discussed. It was necessary for the patients to understand the relationships between the fear/worry, obsessions, and compulsions (overt or covert). Also, the patients should understand that they would not suffer from OCD symptoms if they were not affected by the FEAR/WORRY of the negative events. Then, they were taught mainly to use appraisal-focused coping (e.g. denial, acceptance, redefinition, or logical analysis) to cope with the fear/worry.

Step 3 was to cope with obsessions, but without using exposure and response prevention (ERP). After the intensity of the fear/worry was reduced, the logical step involved how to deal with obsessions. In CCT, an obsession was considered nothing but invoking a fear/worry of negative events. Also, based on the understanding that the more efforts were made to respond to the obsessions, the more frequently and intensely the obsessions or fear/worry were experienced, and that the obsessions had nothing to do with the occurrence of negative events, the patients were taught to using coping strategies in order to disregard the obsessions in their mind, but not to respond to (e.g. try to control or even increase their willingness to accept) them. That will reduce effects of obsession or fear/worry on patients with OCD.

Step 4 was to cope with urge to perform neutralizing behaviors (e.g. sublimation) in order to eliminate over or covert compulsions. Besides disregarding the urge of performing compulsions (leaving it alone), the patients were taught to do something else that had nothing to do with their obsessions. Individuals with OCD practiced proper coping strategies at least three times under the guidance of a therapist. In step 4 of CCT the intensity of anxiety was not increased, but immediately decreased.

**Diagnostic and Assessment Interview**

Diagnosis of OCD according to DSM-IV criteria was made by two experienced senior psychiatrists after face-to-face interview. The symptoms of OCD were evaluated using the Yale-Brown Obsessive Compulsive Symptom Checklist before undergoing CCT. OCD symptom severity was evaluated using the Y-BOCS before treatment and after treatment at week 4.

**RESULTS**

Supplementary TABLE 1. Demographic and clinical characteristics of the participants

|  | CCT  (n = 19) | SSRI+CCT  (n = 19) | SSRI  (n = 21) | F, t, or χ^2^ | *P* value |
| --- | --- | --- | --- | --- | --- |
| Age, years ^a^ | 27.2 ± 5.7 | 28.1 ± 5.1 | 30.1 ± 11.1 | F=1.14 | 0.327 |
| Age at OCD onset, years ^a^ | 20.1 ±6.6 | 21.2 ± 7.2 | 23.5 ± 9.8 | F=1.30 | 0.282 |
| Duration of illness, years ^a^ | 7.6 ± 5.5 | 8.3 ± 4.6 | 7.8 ± 7.2 | F=0.14 | 0.874 |
| Education, years ^a^ | 12.5 ± 3.2 | 13.9 ± 3.0 | 12.5 ± 2.9 | F=0.09 | 0.911 |
| Y-BOCS score ^a^ | 26.4 ± 5.1 | 25.3 ± 6.0 | 26.0 ± 5.4 | F=0.16 | 0.852 |
| Gender, No. (%) |  |  |  |  |  |
| Male | 10 (52.6) | 12 (63.2) | 10 (47.6) | χ^2^=1.0 | 0.607 |
| Female | 9 (47.4) | 7 (36.8) | 11 (52.4) |  |  |
| Marital status, No. (%) |  |  |  |  |  |
| Single | 10 (52.6) | 7 (36.8) | 9 (42.9) | χ^2^=0.98 | 0.613 |
| Married | 9 (47.4) | 12 (63.2) | 12 (57.1) |  |  |
| Symptom of OCD, No. (%) |  |  |  |  |  |
| Obsessions |  |  |  |  |  |
| Contamination | 5 (26.3) | 7 (36.8) | 7 (33.3) | χ^2^=0.50 | 0.771 |
| Aggressive | 3 (15.8) | 2 (10.5) | 2 (9.5) | Fisher’s exact | 0.810 |
| Sex | 1 (5.3) | 1(5.3) | 3 (14.3) | Fisher’s exact | 0.492 |
| Religion | 0 (0.0) | 1 (5.3) | 3 (14.3) | Fisher’s exact | 0.190 |
| Hoarding | 1 (5.3) | 0 (0.0) | 0 (0.0) | Fisher’s exact | 0.343 |
| Pathological doubt | 9 (47.4) | 11 (57.9) | 7 (33.3) | χ^2^=2.45 | 0.293 |
| Symmetry | 2 (10.5) | 1 (5.3) | 1 (4.8) | Fisher’s exact | 0.731 |
| Other | 5 (26.3) | 2 (0.0) | 1 (4.8) | Fisher’s exact | 0.124 |
| Compulsions |  |  |  |  |  |
| Washing | 5 (26.3) | 5 (26.3) | 7 (33.3) | χ^2^=0.33 | 0.850 |
| Checking | 7 (36.8) | 9 (47.4) | 7 (33.3) | χ^2^=0.88 | 0.644 |
| Repeating rituals | 8 (42.1) | 6 (31.6) | 3 (42.9) | Fisher’s exact | 0.145 |
| Hoarding | 0 (0.0) | 0 (0.0) | 0 (0.0) |  |  |
| Order | 5 (26.3) | 3 (15.8) | 1 (4.8) | Fisher’s exact | 0.166 |
| Medication dose, mg/d, mean (SD) |  |  |  |  |  |
| Clomipramine ^b^ |  | 191.1 (85.2) | 193.4 (84.2) | t=0.01 | 0.99 |

a: mean ± SD

b: SSRIs were calculated to the dose equivalence of clomipramine.

Supplementary TABLE 2. Demographic and clinical characteristics of the participants

|  | Response  (n =15) | Remission  (n =23) | t or χ^2^ | *P* value |
| --- | --- | --- | --- | --- |
| Age, y, mean (SD) | 27.6 (5.1) | 29.7 (5.7) | t=1.16 | 0.255 |
| Age at OCD onset, y, mean (SD) | 19.0 (4.9) | 21.7 (7.1) | t=1.28 | 0.207 |
| Duration of illness, y, mean (SD) | 7.6 (7.1) | 9.0 (4.6) | t=0.74 | 0.464 |
| Education, y, mean (SD) | 11.5 (2.9) | 13.0 (3.0) | t=1.53 | 0.135 |
| Y-BOCS score, mean (SD) | 25.3 (6.0) | 26.4 (5.1) | t=0.61 | 0.548 |
| Gender, No. (%) |  |  |  |  |
| Male | 10 (66.7) | 15 (65.2) | χ^2^=0.01 | 0.929 |
| Female | 5 (33.3) | 8 (35.8) |  |  |
| Marital status, No. (%) |  |  |  |  |
| Single | 8 (53.3) | 13 (56.5) | χ^2^=0.04 | 0.847 |
| Married | 7 (47.7) | 10 (43.5) |  |  |
| Symptom of OCD, No. (%) |  |  |  |  |
| Obsessions |  |  |  |  |
| Contamination | 5 (33.3) | 7 (30.4) | χ^2^=0.04 | 0.851 |
| Aggressive | 2 (13.3) | 3 (13.0) | Fisher’s exact | 0.668 |
| Sex | 0 (0.0) | 2 (8.7) | Fisher’s exact | 0.359 |
| Religion | 0 (0.0) | 1 (4.3) | Fisher’s exact | 0.605 |
| Hoarding | 0 (0.0) | 1 (4.3) | Fisher’s exact | 0.605 |
| Pathological doubt | 5 (33.3) | 11 (47.8) | χ^2^=0.30 | 0.583 |
| Symmetry | 1 (6.7) | 2 (8.7) | Fisher’s exact | 0.660 |
| Other | 1 (6.7) | 4 (17.4) | Fisher’s exact | 0.332 |
| Compulsions |  |  |  |  |
| Washing | 4 (26.7) | 6 (26.1) | Fisher’s exact | 0.626 |
| Checking | 5 (33.3) | 11 (47.8) | χ^2^=0.30 | 0.583 |
| Repeating rituals | 3 (20.0) | 11 (47.8) | Fisher’s exact | 0.080 |
| Hoarding | 0 (0.0) | 1 (4.3) | Fisher’s exact | 0.605 |
| Order | 2 (13.3) | 6 (26.1) | Fisher’s exact | 0.302 |

Supplementary Table 3. Regions where the ALFF were significantly changed after CCT and pCCT

| Regions | BA | Number of voxels | Peak activation strength (t) | Peak coordinates | | |
| --- | --- | --- | --- | --- | --- | --- |
|  |  |  |  | x | y | z |
| Post- < Pre- treatment  Insula_L  Putamen_L,  Postcentral_L | 13 | 65 | -4.5123 | -30 | -12 | 9 |
| Post- > Pre- treatment  Occipital_Mid_R,  Occipital_Inf_R,  Lingual_R | 18/19 | 76 | 4.2012 | 36 | -93 | 6 |

A pared-sample t-test showed that there were significantly different ALFF in two clusters of brain regions when compared between pre- and post-treatment in patients with OCD.

BA denotes Brodmann’s area.

References

Callan, V. J., & Hennessey, J. F. (1989). Strategies for coping with infertility. *British Journal of Medical Psychology, 62 ( Pt 4)*, 343-354.

Folkman, S., & Lazarus, R. S. (1980). An analysis of coping in a middle-aged community sample. *Journal of Health and Social Behavior, 21*(3), 219-239.

Hu, X.-Z. (2010). [A Novel Cognitive-Coping Therapy for Obsessive-Complusive Disorder]. *J Applied Clinical Pediatrics, 25*(24), 1848-1851.

Hu, X.-Z. (2012). *[Obsessive-Compulsive Disorder Congnitive-Coping Therapy Mannual]*. Xian City: Xian Jiaotong University Press.

Hu, X.-Z., & Ma, J.-D. (2011). [Clinical effect of pharmacotherapy combined with cognitive-coping therapy on obsessive-compulsive disorder]. *Journal of Xinxiang Medical College, 28*(1), 68-72.

Hu, X. Z., Ma, J. D., Huang, P., Shan, X. W., Zhang, Z. H., Zhang, J. H., . . . Wang, C. H. (2015). Highly efficacious cognitive-coping therapy for overt or covert compulsions. *Psychiatry Res, 229*(3), 732-738. doi:10.1016/j.psychres.2015.08.010

Hu, X. Z., Wen, Y. S., Ma, J. D., Han, D. M., Li, Y. X., & Wang, S. F. (2012). A promising randomized trial of a new therapy for obsessive-compulsive disorder. *Brain Behav, 2*(4), 443-454. doi:10.1002/brb3.67

Lazarus, R. S., & Folkman, S. (1984). *Stress, appraisal, and coping*. New York: Springer Publishing Company.

Ma, J. D., Wang, C. H., Li, H. F., Zhang, X. L., Zhang, Y. L., Hou, Y. H., . . . Hu, X. Z. (2013). Cognitive-coping therapy for obsessive-compulsive disorder: a randomized controlled trial. *J Psychiatr Res, 47*(11), 1785-1790. doi:10.1016/j.jpsychires.2013.08.002
